# Utilizing DeepSqueak for automatic detection and classification of mammalian vocalizations: a case study on primate vocalizations

**DOI:** 10.1038/s41598-021-03941-1

**Published:** 2021-12-27

**Authors:** Daniel Romero-Mujalli, Tjard Bergmann, Axel Zimmermann, Marina Scheumann

**Affiliations:** 1 Institute of Zoology, University of Veterinary Medicine Hannover, Bünteweg 17, 30559 Hannover, Germany; 2 University of Aalen, Aalen, Germany

**Keywords:** Behavioural ecology, Classification and taxonomy, Communication and replication, Machine learning, Animal behaviour

## Abstract

Bioacoustic analyses of animal vocalizations are predominantly accomplished through manual scanning, a highly subjective and time-consuming process. Thus, validated automated analyses are needed that are usable for a variety of animal species and easy to handle by non-programing specialists. This study tested and validated whether DeepSqueak, a user-friendly software, developed for rodent ultrasonic vocalizations, can be generalized to automate the detection/segmentation, clustering and classification of high-frequency/ultrasonic vocalizations of a primate species. Our validation procedure showed that the trained detectors for vocalizations of the gray mouse lemur (*Microcebus murinus*) can deal with different call types, individual variation and different recording quality. Implementing additional filters drastically reduced noise signals (4225 events) and call fragments (637 events), resulting in 91% correct detections (N_total_ = 3040). Additionally, the detectors could be used to detect the vocalizations of an evolutionary closely related species, the Goodman’s mouse lemur (*M. lehilahytsara*). An integrated supervised classifier classified 93% of the 2683 calls correctly to the respective call type, and the unsupervised clustering model grouped the calls into clusters matching the published human-made categories. This study shows that DeepSqueak can be successfully utilized to detect, cluster and classify high-frequency/ultrasonic vocalizations of other taxa than rodents, and suggests a validation procedure usable to evaluate further bioacoustics software.

## Introduction

Animal bioacoustics is a growing field in basic and applied research. One central research topic is the development of bioacoustic monitoring systems (e.g., Refs.^1–5^) to improve animal management by monitoring animal welfare, animal health, animal behavior and animal reproductive state (e.g., Refs.^6–12^) or to monitor animal abundance for animal conservation (e.g., Refs.^13–18^). In the last decades, animal bioacoustic research especially in the ultrasonic range has been largely limited by technical issues such as frequency characteristics of the microphone, sampling rates, working memory of the recorder or storage capacity. However, these technical hurdles have been overcome by recent technological advancement. Thus, today, researchers are able to record animal vocalizations, even in the ultrasonic range, 24 h per day for several days per week (e.g., Song Meter model SM4/SM4BAT-FS (Wildlife Acoustics Inc., Maynard, MA, USA), AURITA^19^). Therefore, today in animal vocalization research, bioacoustic analysis of vocal recordings is the limiting factor and not technical limitations of the recording.

The traditional method used for screening vocal recordings is visual scanning and classification of vocalizations based on the sonogram^20,21^. However, manual scanning and classification are unfeasible for collecting thousands of vocalizations. Additionally, the success of this approach often depends on the knowledge and experience of the analyst^21^. Thus, we need new automatic analysis tools to speed up the screening of these large data streams, and to detect and classify call types independently from the experience of the human observer. Animal bioacoustics researchers have developed several detection systems for animal vocalizations using mathematical algorithms (e.g., Refs.^3,4,11,22^). Although the usage of these systems is widespread in specific subgroups of the bioacoustic community depending on the animal group of interest (e.g., PAMGuard in the marine mammal community^22^, Kaleidoscope in the bat community^23^, MUPET in the rodent community^4^), these rarely overcome the taxa boundaries so that they can be used by the whole bioacoustic community including small and large bodied terrestrial, aquatic mammals and birds. One reason is that animal vocalizations vary largely in the frequency range (from infrasound to ultrasound) and in their acoustic structure (harmonic to noisy calls), making it difficult to develop a general animal vocalization recognition system. Thus, most tools were designed for a specific animal species and/or vocalization types (e.g., birds^1,24^, rodents^2,25^, primates^26–31^, cetacea^3,32,33^, elephants^34,35^, pigs^9,11,12^, bats^36,37^). Another reason is that these programs are difficult to master for non-specialists because some of these tools require a fundamental understanding of bioacoustics and programing. Thus, software is needed which can be used for a wide variety of animal species and is easy to handle for non-specialists.

In 2019, DeepSqueak^25^, a promising new software suited for automatic detection, classification and clustering of ultrasonic vocalizations (USV) was introduced to the animal bioacoustic community. While originally built with focus on ultrasonic mice and rat vocalizations ranging from 20 to 115 kHz, the DeepSqueak GUI (graphical user interface) is intuitive and parameters can be adjusted for other species without pre-required skills in programing. The core feature of DeepSqueak is its state of the art application of a machine-learning algorithm. The algorithm is similar to convolutional neural networks (CNN) used for automatic speech recognition systems such as Alexa, Siri or Cortana^38,39^. Using faster regional-convolutional neural networks (Faster-RCNN)^40^, DeepSqueak has an increased call detection rate, a reduction in the number of false positives, a reduction in analysis time compared to older detection software (such as Ultravox (Noldus, Wageneing, NL)^5^, MUPET^4^ or Xbat^41^). DeepSqueak allows multiple Faster-RCNN centered approaches for automatic USV detection. In addition, DeepSqueak has a GUI that enables users to conduct manual reviewing, editing and labeling of detection files. It also integrates supervised classification networks and unsupervised clustering models that can be trained and used for further analyses of data. Supervised classification networks can be trained based on detections labeled according to, for example, user pre-defined clusters (call types). In the unsupervised approach, these clusters are created based on frequency, shape parameters and duration of vocalizations either by the k-means or by dynamic time-warping (experimental algorithm) method. The supervised approach has the advantage to automate, and therefore speed up the identification of the vocal repertoire of a species based on information from previously described call categories^27^. The unsupervised model has a less controllable outcome but is unbiased to priori assumptions of the observer.

**Figure 1:**
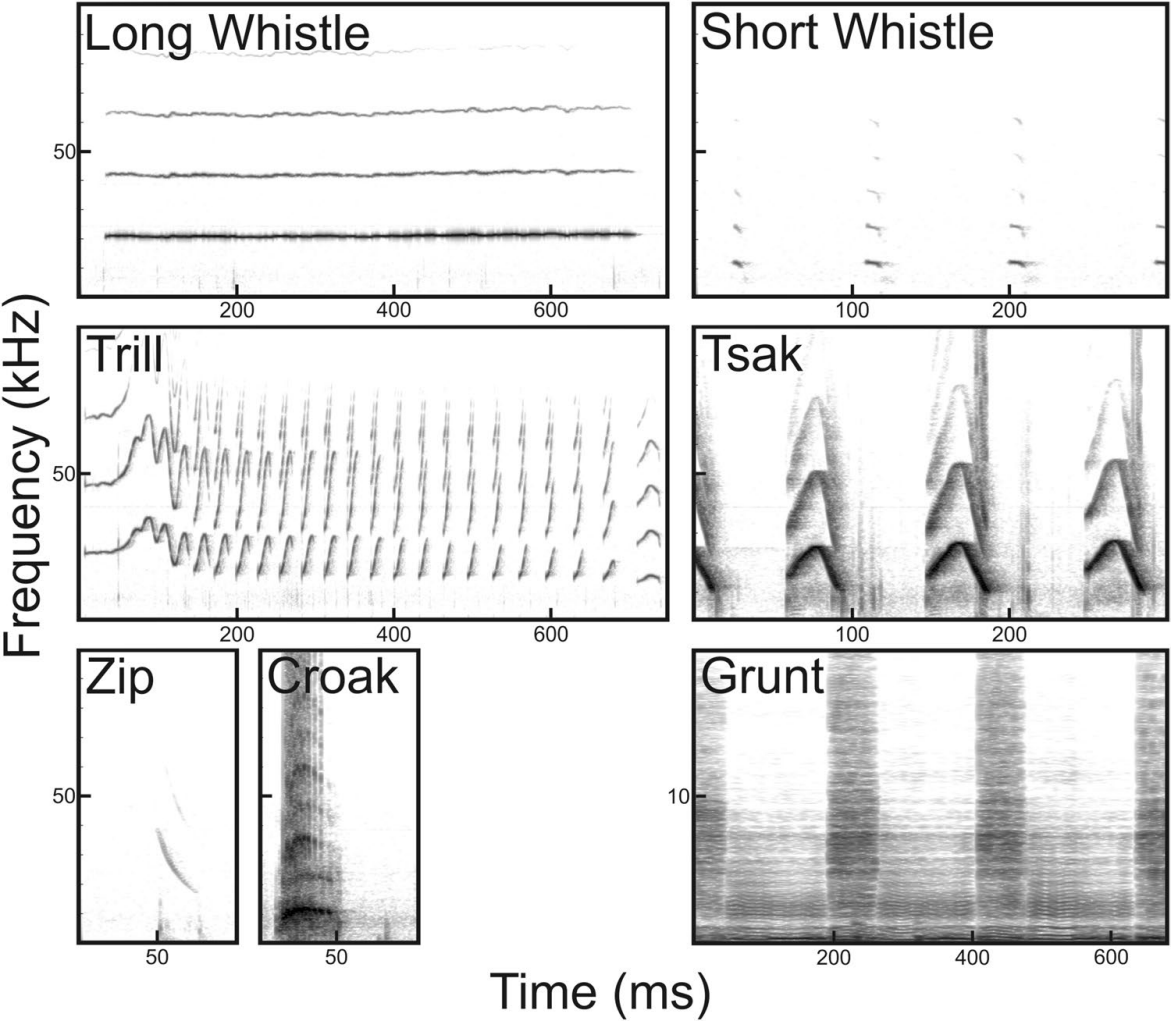
Vocal repertoire of the gray mouse lemur (*M. murinus)* based on Zimmermann (2018)^49,50^.

In the present study, we tested whether DeepSqueak can be utilized to analyze high-frequency to ultrasonic vocalizations in a primate species, the gray mouse lemur (*Microcebus murinus*), and whether the detector’s ability is sufficiently general to be used for other mouse lemur species. Mouse lemurs are nocturnal strepsirrhine primates endemic to the dry forest of Madagascar^42^. They are able to produce calls and to perceive acoustic information from audible to ultrasonic frequency range (auditory frequency range: 800 Hz to 50 kHz^43,44^; voice production range: 400 Hz to 30 kHz of the fundamental frequency^45–47^). The best studied species is the gray mouse lemur for which to date ten call types are described (e.g., Refs.^48–50^). Seven call types (Grunts, Croaks, Tsaks, Long whistles, Short whistles, Zips and Trills, Fig. 1) are typical for the adult vocal repertoire. Thereby, Grunts and Croaks are low-frequency noisy calls, whereas Tsaks, Short whistles, Long whistles, Trills and Zips are harmonic calls uttered in the high-frequency to ultrasonic range and differ in the contour of the fundamental frequency. Tsaks and Short whistles are uttered in series of variable duration during agonistic or alarm situations, whereas Long whistles, Trills and Zips are uttered singly. Long whistles are uttered during sleeping group formation and mating mainly by females. Trill is the acoustically most complex call consisting of several syllables, uttered during various social interactions (e.g., mating^51^, mother-infant reunions^52^ and sleeping group formation^53^). Zips are soft calls often associated with Trills, whose social function remains uncertain. Mouse lemur calls carry indexical cues encoding kinship^54,55^, familiarity (dialects^56^), individual identity^47,57^ and hormonal status^58^.

The aim of our study was to test whether DeepSqueak developed for mouse and rat vocalizations can be utilized to detect, cluster and classify vocalizations of a primate, the gray mouse lemur, uttered in the high-frequency to ultrasonic range. Additionally, we investigated whether detectors trained for *M. murinus* can also be used to detect vocalizations of a closely related mouse lemur species, *M. lehilahytsara.* Defining call types through visual inspection is especially difficult when differences among call types are not discrete but continuous^59,60^ (e.g., transitions from Short whistle to Tsak). Thus, we tested whether supervised classifier can be used to objectively label mouse lemur vocalization. Additionally, we explored to which extent clusters of mouse lemur vocalization defined by the algorithm (unsupervised model) match the already established human-based call type categories reported in the literature^48–50^.

## Methods

### Data sets and preparation

The data sets used in this study originated from the sound archives of the Institute of Zoology, University of Veterinary Medicine Hannover, Germany storing sound recordings from the captive self-sustaining mouse lemur breeding colony of the institute (license: AZ 33. 12-42502-04-14/1454; AZ33. 19-42502-11A117). All recordings were made in a sound-attenuated room at the Institute of Zoology. The sound recordings were obtained during various independent behavioral studies (e.g., social encounter paradigm, playback study^43,47^) conducted between 2003 and 2012 using recording equipment sensitive to the high-frequency to ultrasonic range (see Supplementary Table S1 for details on the experimental paradigms and recording equipment). We used only vocalizations from adult animals, since these vocalizations are well studied (e.g., Refs.^49,50^). Details on the acoustic parameters of the studied call types can be found in Supplementary Tables S2 and S3.

For the training data set, we used 2123 vocalizations belonging to the five most common call types to represent the high-frequency/ultrasonic adult vocal repertoire^49,50^. The chosen types range from short to long calls, from unmodulated to highly modulated calls, as well as weak to strong amplitude vocalizations providing a great insight into the general performance of DeepSqueak to identify diverse syllable structures as well as detection performance on amplitude variation rich syllable regions and calls. The training data set was obtained from 27 preselected recording sessions and recorded from 24 individuals or groups (in the case where the sender could not be reliably determined, because the observer could not hear and spatially locate the sender of the ultrasonic call). The recording sessions originated from the social encounter, the handling and the mother-infant reunion paradigm (Supplementary Table S1) and included different recording qualities to increase the robustness of the detector against individual variation in the call structure and differences in the recording quality. To validate the detectors, we used the standardized and experimental data sets (evaluation data sets; Table 1).

**Table 1:** Description of the training, standardized and experimental data sets used in this study. The table represents the number of calls for each call type included in the respective data set. *N*_*calls*_ number of calls, *N*_*Ind/groups*_ number of individuals or groups from which the calls were emitted.

Data set	Trill		Long whistle		Short whistle		Tsak		Zip	
	N_calls_	N_Ind/groups_	N_calls_	N_Ind/groups_	N_calls_	N_Ind/groups_	N_calls_	N_Ind/groups_	N_calls_	N_Ind/groups_
**Training data set**										
Detection (2123 calls)	326	12	159	4	1109	6	519	5	10	1^a^
Classification and clustering (2257 calls)	302	52	186	24	1158	46	541	34	70	20
**Standardized data set**										
Good-quality (450 calls)	50	42	50	22	150	40	150	29	50	19
Clipped (80 calls)	10	9	10	8	30	9	30	10	–	–
Low-amplitude (80 calls)	10	7	10	10	30	10	30	10	–	–
Overlaid (80 calls)	10	8	10	8	30	8	30	9	–	–
**Original experimental data set**										
*M. murinus* (3040 calls)	76	7	157	5	2582	8	195	6	30	7
*M. lehilahytsara* (1115 calls)	48	5	526	5	240	1	193	3	108	4

^a^Zips occur rarely in a sufficient signal-to-noise ratio. However, because they are highly stereotyped, this low number of Zips turned out to be sufficient to train the detector.

**Figure 2:**
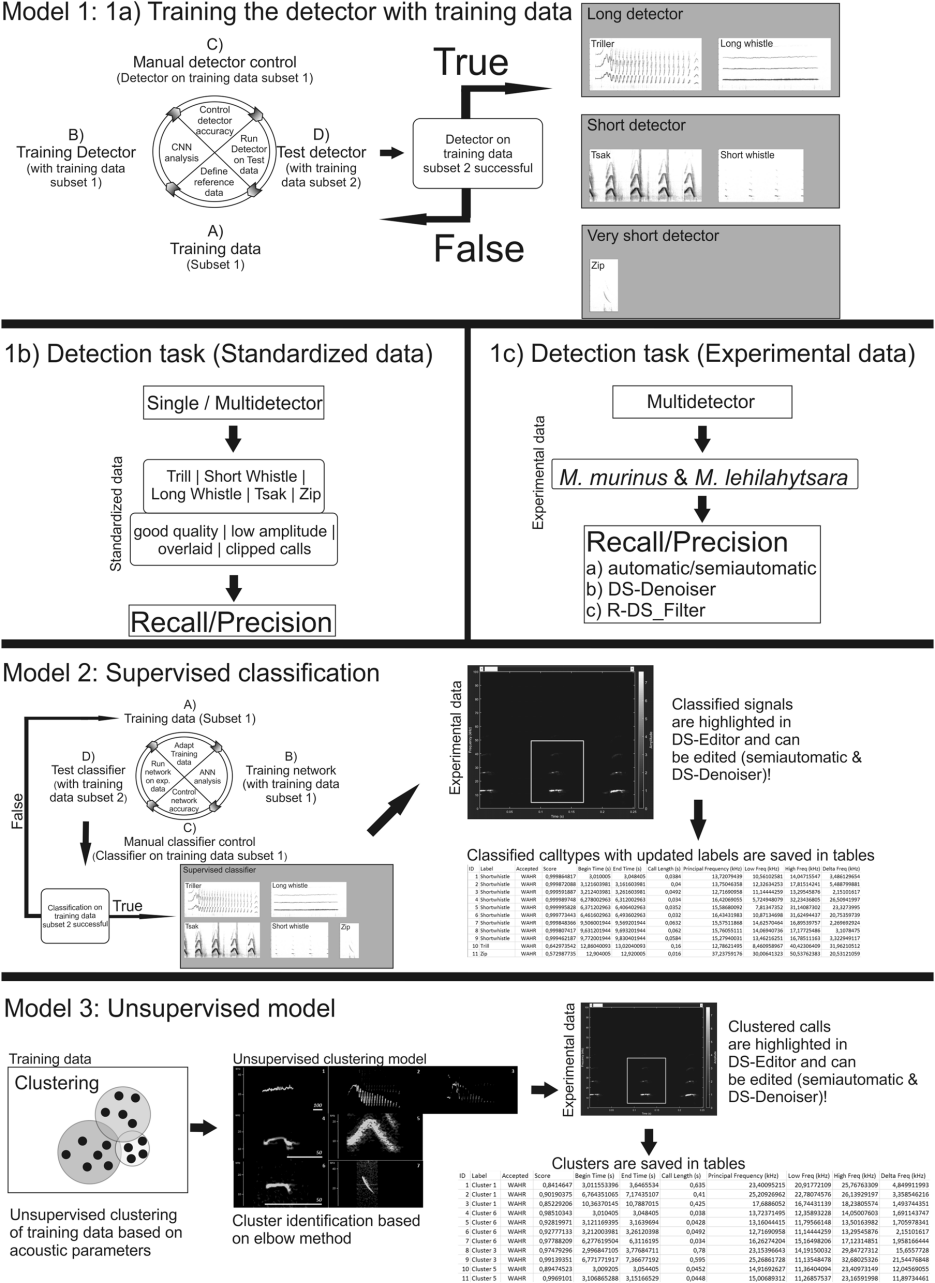
Scheme illustrating the three different models trained and evaluated in this study. Model (1) Detection task: (1a) Training the detectors using image-based CNN with the training data. Evaluation of the trained detectors with (1b) the standardized data and (1c) the experimental data. Model (2) Supervised classification: User defined call type network based on acoustic data. Model (3) Unsupervised model: k-means model based on acoustic data.

The standardized data sets were used to test for the robustness of individual variation and recording quality (Fig. 2: 1b; for detailed information, see Supplementary Methods: Additional information on the preparation of the standardized data sets). The term standardized refers to the fact that we combined single vocalizations (not used in the training data set) of the sound archive to a single audio file to maximize the number of different individuals/groups for the five trained call types (Long whistle, Trill, Zip, Short whistle, Tsak) and to test four different recording quality scenarios: good-quality, clipped, low-amplitude, overlaid calls (Table 1; for the definition of the quality scenarios, see Supplementary Methods: Additional information on the preparation of the standardized data sets). Whereas Long whistles, Trills and Zips are emitted singly, Tsaks and Short whistles are emitted in a series, ranging from five up to multiple hundred calls. To represent the natural structure of Tsaks and Short Whistles three continuous calls of a series were selected (as a compromise between real life occurrence and data evaluation) to test whether the detectors could detect the single calls within a series, which are separated by a short intercall-interval (< 200 ms)^61^. For each call type, the single calls/series were combined to a single audio file to test whether performance was consistent across different call types, using PRAAT (www.praat.org)^62^ combined with GSUPraatTool 1.9^63^ (for detailed information, see Supplementary Methods: Additional information on the preparation of the standardized data sets). As original audio data contained intercall-intervals of background noise between calls, we added three seconds of white noise with an amplitude of 28 db between two consecutive calls that were combined into standardized data sets to simulate real life noise. For the good-quality standardized data set, 50 single calls/series were combined for each call type. For the clipped, low-amplitude and overlaid standardized data sets, ten single calls/series were used for each call type (Table 1). For Zips, there were only data available for the good-quality standardized data set.

The experimental data set was used to investigate the performance of the detector on original experimental audio data (Fig. 2: 1c) recorded in social encounter experiments of *M. murinus* (ten recording sessions = 140.11 min), which can contain multiple call types and background noise. To investigate whether the detectors can also be used to detect calls of an evolutionary closely related mouse lemur species, we selected ten recording sessions (50 min) recorded during social encounter experiments from *M. lehlilahytsara* (Table 1).

### Procedures in the DeepSqueak program

DeepSqueak^25^ (version 2.0; download link https://github.com/DrCoffey/DeepSqueak) was used on MATLAB (version 2018a). DeepSqueak mainly integrates three machine-learning models: Model (1) Faster regional convolutional neural network (Faster-RCNN), which detects signals of interest from filtered sonograms after applying a series of convolutions and filters (Detection task), Model (2) An acoustic-based classification neural network that classifies unlabeled data based on the spectral structure and duration of the calls (Classification task) and Model (3) An acoustic-based k-means unsupervised clustering algorithm (Clustering task). The program integrates a method of robust contour detection, which consists in finding the frequency at maximum amplitude for each time point, and then cleaning the spectrogram by removing non-tonal features based on the concept of tonality (for more details see Ref.^25^). Based on the contour detection, temporal, spectral and tonality-related acoustic parameters were extracted automatically from the respective contour (see definition of the acoustic parameters in Supplementary Table S2).

In the following, we performed three different models (Fig. 2): Using model 1 we trained the detectors with the training data set. For the training procedure, DeepSqueak automatically generated two sub data sets a learning sub set and a testing sub set in a ratio of 8:2. When the detector reached high identification accuracy within the learning sub set, the detectors were tested on the testing subset (Fig. 2: 1a). Only when both subsets reached highest identification accuracy with the appropriate detectors, these detectors were tested using the evaluation data set (standardized and experimental data set, Fig. 2: 1b + 1c). Using model 2, we tested the acoustic-based supervised classification of call types and using model 3 we explored to which extent the unsupervised classification matches the already published call type categories^49,50^. Thereby, model 1 is using a convoluted neuronal network based on image data (Fig. 2: 1a) to create Faster-RCNN-based detectors. In contrast, model 2 and 3 use only audio parameters to create a classifier and an unsupervised model (Fig. 2: 2 + 3). The supervised classifier creates an acoustic-based neural network using the spectral structure and duration of reference calls, while the unsupervised model creates audio-based clusters calculated by the k-mean algorithm. In both scenarios (supervised/unsupervised approach) all audio data is cumulatively analysed by DeepSqueak, therefore, using individual short audio files or merged audio data make no difference when creating audio-based networks in DeepSqueak.

#### Model 1: Detection task

##### Training of the detectors

The training of more than one detector was recommended by the DeepSqueak authors when call duration differs between call types^25^. In our test scenario, we manually sorted the calls in three duration-based groups (Long duration = Long Whistle & Trills; Short duration = Short Whistle & Tsak; Very Short duration = Zip; for detailed acoustic information, see Supplementary Tables S2 and S3). Therefore, we trained three detectors based on the training data: Long detector, Short detector, Very short detector (Fig. 2: 1a). The training of the faster RCNN detectors was based on a total of 4536 images (for detailed information, see Supplementary Method: Additional information on the settings of the training).

##### Evaluation of the detectors

We validated the trained detectors by using the standardized and experimental data sets. The standardized data sets (Fig. 2: 1b) were used to test the robustness of the detector to individual variation in the acoustic structure of the calls as well as different recording problems affecting the quality in the audio recordings (good-quality, clipped, low-amplitude or overlaid calls). The experimental data sets (Fig. 2: 1c) were used to test how original data containing mixed call types were detected and to investigate the cross-taxa capacity of the detector. Thus, we investigated whether the detector can also be used to detect high-frequency to ultrasonic vocalizations of a closely related mouse lemur species, *M. lehilahytsara*.

All audio files of the standardized and the experimental data sets used in this study were screened by the algorithm in the frequency range of 5 to 50 kHz (for detailed information, see Supplementary Methods: Additional information on the procedure and setting of the detection task). To test whether the single detectors work as intended and can deal with individual variation of the calls, we tested the performance of the single detectors for all five call types using the good-quality standardized data set. We predicted that for Long whistles and Trills, the Long detector should show the best performance; for Short whistles and Tsaks, the Short detector; and for Zips, the Very short detector.

To estimate how robust the intended detectors are against variation in recording quality (clipped, low-amplitude, overlaid calls), we compared the performance of the respective detectors between standardized data sets of the good-quality, clipped, low-amplitude and overlaid scenario.

The detectors could be selected individually (single detector) or simultaneously (multi-detector) within the DeepSqueak GUI if scientists are interested in detecting various call types at once. To test the performance of combined detectors using all three detectors simultaneously (multi-detector), we used the good-quality standardized data sets. Preliminary analysis showed that the simultaneous use of multiple detectors increases the amount of false positives (personal observation). This occurs because the Short and the Very short detector identifies single syllables of the Trill or various call fragments of the same Long whistle as single calls. To filter these doublet syllables and to merge fragments of a call, a function in R (v3.5.2^64^; termed R-DS Filter) was developed and applied (for detailed information, see Supplementary Methods: Additional information on the R-DS Filter).

To test the detectors under real life condition, we used the experimental data set of *M. murinus*, which contain various call types co-occurring in the same recording and which are often accompanied by background noise (e.g., scratching, digging of the animals). To reduce the effect of background noise, DeepSqueak offers the possibility to train a denoiser network (DS-Denoiser) to remove unwanted noise signals from detections. For this, 598 typical noise events (negative training samples) and 891 USV (positive training samples) were manually marked as correct or false detections in the DeepSqueak GUI and used to train the denoiser network. The confusion matrix based on a subsample of the training data showed that 62 of 63 noise events (98%) and 86 of 86 USV events (100%) were correctly classified (Supplementary Fig. S1). After applying the DS-Denoiser, the output files were run through the R-DS Filter to minimize duplicates caused by simultaneously using the three detectors (see above).

#### Model 2: Supervised classification

We trained an acoustic-based supervised network based on the training data and the standardized data set of good-quality calls, and tested the performance of the supervised network to classify gray mouse lemur vocalizations using real data obtained from social encounter experiments (experimental data set; Fig. 2: 2). The data used to train the classifier network consisted of a total of 2257 representative calls (manually labeled by DRM) of good-quality selected from the previous detection training data set and the good-quality standardized data set (for detailed information, see Supplementary Methods: Additional information on the procedure of the classification). The confusion matrix based on a subsample revealed that 20 of 21 Long whistles (95%), 28 of 28 Trills (100%), 100 of 100 Short whistles (100%), 43 of 43 Tsaks (100%), four of four Zips (100%) were correctly classified (Supplementary Fig. S2).

To test the performance of the supervised network, the supervised classifier was applied to unlabeled detection files from the experimental data set of *M. murinus*.

#### Model 3: Unsupervised model—clustering task

We explored the potential of a DeepSqueak built-in acoustic-based unsupervised model to cluster mouse lemur vocalizations into categories defined by the algorithm independently from human intervention. We tested the k-means model already implemented on DeepSqueak to automatically cluster the detected vocalizations into different categories^65^ (Fig. 2: 3). The unsupervised clustering was applied to the same detection files as the supervised classification. Thereby, we set the weighted inputs for the acoustic parameters characterizing the frequency, duration and contour of a call to 1 to have an equal contribution. Furthermore, we let the model automatically decide the optimal number of clusters based on the elbow method^25^ (setting: Max clusters: 50, replicate 100). Afterwards, we compared the obtained clusters (top categories) with the reported call types, already established in the literature for *M. murinus*^49,50^ based on the visual approach (Fig. 1).

### Validation of the detection and classification

**Figure 3:**
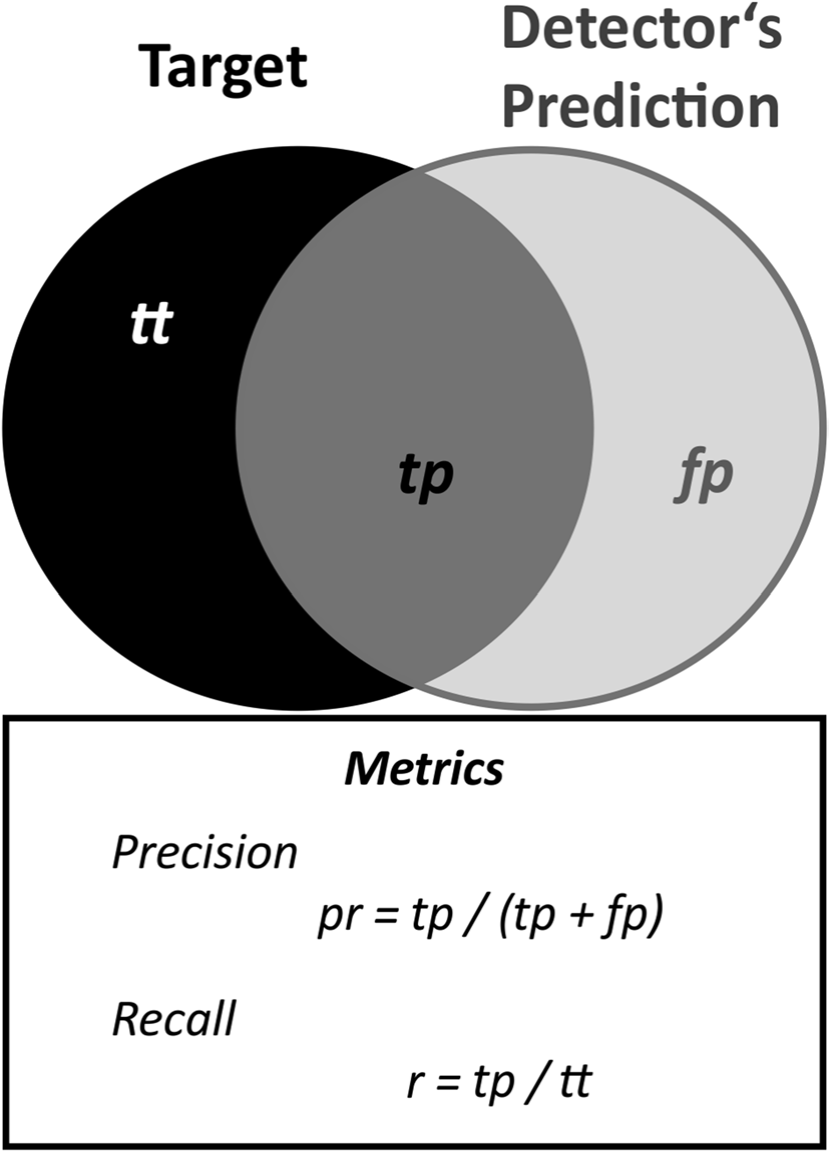
Definition of precision and recall. Metrics used to assess the performance of machine learning models.

For validation of the standardized data sets, we compared the number of calls with the performance of the detectors. For all scenarios, the performance of the detector networks was scored based on their precision and recall, which are common metrics used to evaluate the performance of machine learning models^66^ (Fig. 3). Precision represents the ratio between correct call detection (true positives—*tp*) and mistakes (false positives—*fp*). It is calculated by dividing the number of true positives (hits) by the sum of true positives and false positives (*pr* = *tp*/*(tp* + *fp*). Thus, a value of 1 indicates the best performance (i.e., no mistakes, *fp* = *0*), whereas a value of 0 indicates no single correct detection. Recall represents the ratio between the true positives (*tp*) and the total number of target signals (total true—*tt*) calculated by dividing the true positives by the total number of target signals (*r* = *tp*/*tt)*. Thus, a value of 1 indicates that all target calls were correctly detected, whereas a value of 0 indicates that no single target call was correctly detected.

There were three types of false positive detections: Noise—acoustic signals, which did not belong to any call, Call fragments—parts of the calls, which were detected but not the whole call and Cluster—two or three calls in a series, which were detected as one call. DeepSqueak’s GUI enables users to manually edit the measuring boxes during visual inspection of the detection files and thereby to correct incomplete detections. Thus, we split the assessment of the detection performance into an automated approach and a semi-automated approach. In the automated approach we counted only correctly detected calls as true positive hits without human intervention. This aims at an end-to-end approach. In the semi-automated approach, we manually adjusted the measuring boxes, if possible, when parts of the call were missed (Call fragments) or when calls, typically two up to three elements of a series, were clustered (Cluster). These corrected detections were counted as true positive for the semi-automated approach. This distinction (semi- vs automated approach) allows to assess the potential drop in recall using the end-to-end approach.

To compare the different recording quality scenarios, we calculated the mean of the precision and recall using the respective detector over all call types for both the automated and semi-automated approach. In the overlaid scenario, overlaid calls, two calls of different call types, which were detected as one, were counted as false positive only for the automatic approach.

For the experimental data set of *M. murinus*, we investigated the impact of the DS-Denoiser and the R-DS Filter on the detection performance by calculating the precision and the recall of the detections for each processing step. For validating the experimental data set, we calculated the recall for each recording session and each call type by dividing the number of target calls detected by the detector networks (verified by manual checking of the detection files using the semi-automatic approach) by the number of calls obtained by previous manual scanning of experts in bioacoustics (total true). Thereby, recall values could exceed a value of 1, indicating that the detectors detected more calls than detected by previous manual screening. We calculated the median detection precision and recall as well as the respective interquartile range^67^ (IQR) for the ten recording sessions.

For validating the supervised classifier, we applied the classifier network only to correctly detected target calls (automated approach). Afterwards, we checked the labeling of the supervised classifier manually. To calculate the classifier precision and recall, we used the number of calls, where the label of the detector matched manual labeling as true positives and the number of target signals where the labels did not match as true negatives; the total number of target signals was obtained from the previous manual scanning of bioacoustic experts. We calculated the median classifier precision and recall as well as the respective interquartile range (IQR) for the ten recording sessions.

## Results

### Robustness of individual variation and recording quality using standardized data sets (Model 1)

Using the automated approach, the long calls, Long whistle and Trill, were better detected by the Long detector (Long whistle: precision = 1.0, recall = 0.92; Trill: precision = 0.80, recall = 0.80) than by the Short (Long whistle: precision = 0.66, recall = 0.80; Trill: precision = 0.35, recall = 0.58) and Very short detector (Long whistle: precision = 0.07, recall = 0.22; Trill: precision = 0.02, recall = 0.10) (Supplementary Fig. S3). For the short calls, Short whistle and Tsak, the Short detector performed better (Short whistle: precision = 0.80, recall = 0.57; Tsak: precision = 0.95, recall = 0.89) as compared to the Very short (Short whistle: precision = 0.60, recall = 0.43; Tsak: precision = 0.82, recall = 0.71) and Long detector (Short whistle: precision = 0.18, recall = 0.01; Tsak: precision = 0.75, recall = 0.02). The lower precision for the Short whistle using the Short detector could be explained by the fact that Short whistles of the standardized data set were often clustered. Thus, using the semi-automated approach manually separating the calls into a cluster increased the precision and recall rate to 1.00. The Zip calls were well detected by both the Very short (precision = 0.94, recall = 0.94) and the Short detector (precision = 1.00, recall = 1.00) as compared to the Long detector (recall = 0.00). Thus, the three trained detector networks worked as expected and showed relatively high recall and precision for all call types even if calls of multiple individuals were used.

**Figure 4:**
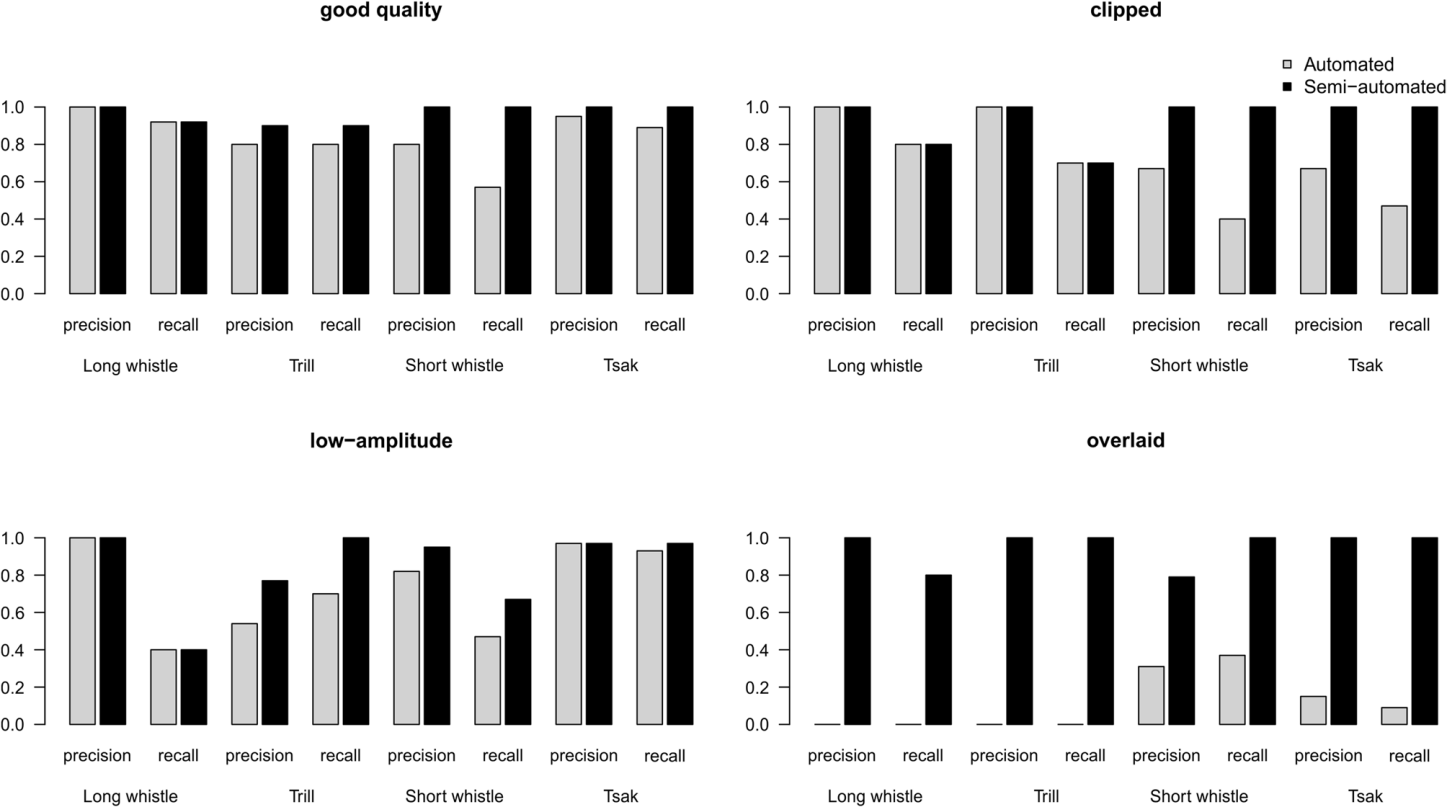
Bar plots of precision and recall of the standardized data sets for each quality scenario (good-quality, clipped, low-amplitude and overlaid signals) for four call types; automated approach indicated in gray; semi-automated approach indicated in black.

To investigate the robustness against quality scenarios, only the best performing detectors for each call type were used (i.e., Long detector for Long whistles and Trills; Short detector for Short whistles and Tsaks; and Very short detector, for Zips). When comparing the detection performance under different quality scenarios using the automated approach, we found a decrease in recall and precision for all scenarios (Fig. 4) compared to the good-quality standardized data set (mean precision = 0.90, mean recall = 0.83). Thereby, detection performance dropped dramatically for the overlaid calls (mean precision = 0.12, mean recall = 0.11), followed by the low-amplitude calls (mean precision = 0.85, mean recall = 0.68) and the clipped calls (mean precision = 0.83, mean recall = 0.59). Under the overlaid scenario, Short whistles and Tsaks were only correctly identified as single detected elements when not all calls within a series were overlaid (Fig. 4). The dropping for the low-amplitude calls could be explained by the weak frequency contours, whereas under the overlaid scenario, target calls were detected mainly together (clustered) with the overlapping calls (Fig. 4). However, using the semi-automated approach, the recall and precision improved for all quality scenarios and were comparable to the good-quality data (mean precision = 0.98, mean recall = 0.96) for the clipped (mean precision = 1.00, mean recall = 0.88), the low amplitude (mean precision = 0.94, mean recall = 0.80) and overlaid scenarios (mean precision = 0.95, mean recall = 0.95).

### Multi-detection, improvement and generalization using standardized and experimental data sets (Model 1)

Using multiple detectors together (multi-detector) for the detection of the good-quality standardized data set, precision dropped dramatically for Long whistles (≤ 0.52) and Trills (≤ 0.28) using both the automated and semi-automated approach (Supplementary Fig. S4). This drop could be explained by the Short and Very short detectors both recognizing call fragments as hits due to amplitude variation within a call, and syllables composing Trills. This problem was solved by applying the self-implemented R-DS Filter deleting overlaying call fragments. After applying the R-DS Filter, the precision increased considerably for Long whistles and Trills using both the automated and the semi-automated approach (Long whistles = 0.96; Trill ≥ 0.73) (Supplementary Fig. S4). In contrast, using the automated approach, for Short whistles and Tsaks precision (≤ 0.77) and recall (≤ 0.57) dropped when using multi-detection combined with the R-DS Filter. This was due to the fact that the calls within a series were clustered. Editing the measuring boxes during the visual inspection (semi-automated approach) improved the precision and recall for these two call types (precision ≥ 0.99, recall = 1.00).

**Figure 5:**
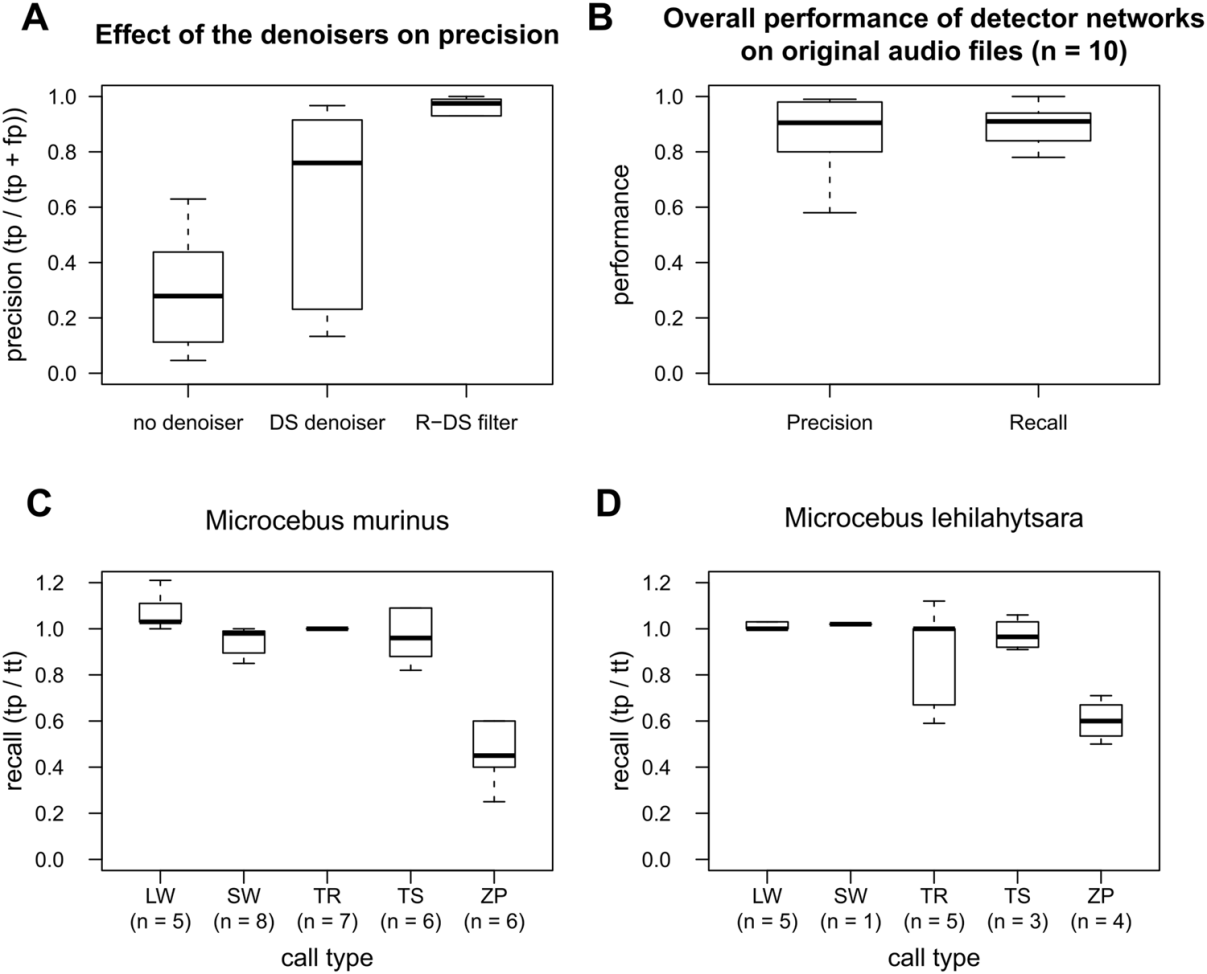
Boxplots of performance of the multi-detector using the experimental data set; (**A**) Performance during each step of the analysis (first step: detection; second step: application of the DS-Denoiser to remove undesired background noise; third step: application of the R script to remove fragments based on the experimental data set of *M. murinus*); (**B**) Overall precision and recall for gray mouse lemurs (*M. murinus)*; (**C**) Detection recall for each call type of vocalizations of *M. murinus* and (**D**) evolutionary closely related species, *M. lehilahytsara*, respectively.

Using the experimental data of *M. murinus*, the DS-Denoiser decreased the false-positive detections considerably (98.55% of the noise was removed). Additionally, the application of the R-DS Filter resulted in a further increase in the precision (median = 0.90, IQR = 0.16) and recall (median = 0.91, IQR = 0.10; Fig. 5a,b). This occurred because the detections contained not only single calls but also call fragments and clusters of more than one target call. Using the semi-automatic approach, the mean recall was comparable across call types, and for four of five call types median recall values greater than 0.96 were achieved (Long whistle: median = 1.03, IQR = 0.11; Trill: median = 1.00, IQR = 0.00; Short whistle: median = 0.98, IQR = 0.07, Tsak: median = 0.96, IQR = 0.18; Fig. 5c). The Zip call had the lowest recall (median = 0.45). This might be explained by the rare occurrence of this call type (0–8 Zip calls per recording). Thus, missing only a single detection can cause a large decrease in the recall value. Additionally, we realized that the detectors detected also 40% of low-frequency vocalizations (Croaks and Grunts counted as false positives in this study), for which they were not trained due to the fact that the ultrasonic recording device was not able to record below 5 kHz.

When testing whether the detector can also be generalized to evolutionary closely related sister species, the Goodman’s mouse lemur (*M. lehilahytsara*), we showed that the detector networks were able to detect most of the calls (recall: median = 0.96, IQR = 0.13; Fig. 5d). For the four call types, which have a similar spectral contour to the respective call types in *M. murinus*, recall values were within the range found for *M. murinus* (Long whistle: median = 1.00, IQR = 0.03; Short whistle: median = 1.02, IQR = 0.00; Tsak: median = 0.96, IQR = 0.09; Zip: median = 0.60, IQR 0.09; Fig. 5c,d). Nonetheless, also the Trill, which differs in the spectral contour from *M. murinus* was well detected (Trill: median = 1.0, IQR = 0.33). However, in this case, the Trill was not detected as one call but as separate two syllables.

### Supervised classification as a tool for automatic call labeling (Model 2)

**Figure 6:**
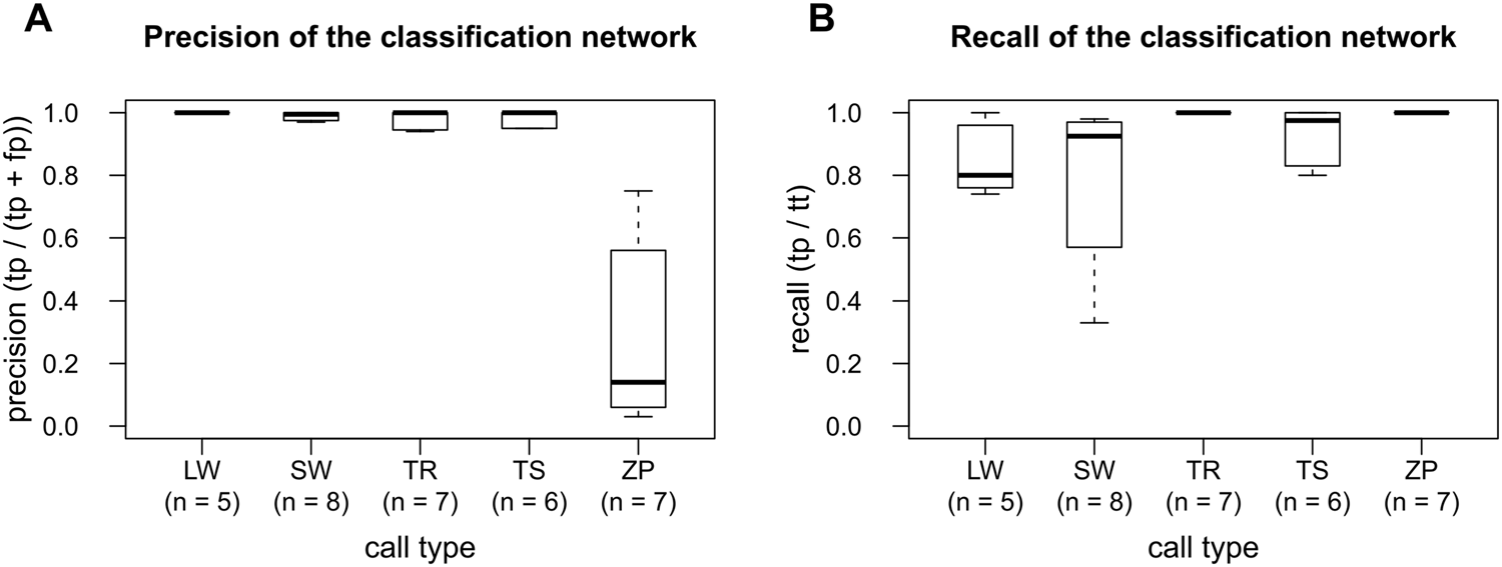
Boxplots of (**A**) precision and (**B**) recall of the supervised classifier labeling the experimental data set of *M. murinus*.

The classifier network automatically assigned the corresponding labels to each call detected in the audio files with a relatively high precision for Long whistles (median = 1.00, IQR = 0.00), Trills (median = 1.00, IQR = 0.05), Short whistle (median = 1.00, IQR = 0.02) and Tsak (median = 1.00, IQR = 0.04). Only for Zips median precision was relatively low (median = 0.14, IQR = 0.50) (Fig. 6a). It is important to notice that Zips were underrepresented compared to other call types in the experimental data set. All detection files had fewer than eight Zips each. Every true Zip was always correctly classified, whereas Short whistles were often misclassified as Zips (Supplementary Table S4). In some cases, the recall for Short whistles was lower than precision due to some misclassifications as Long whistle, Trill, Tsak or Zip (Supplementary Table S4). Similarly, Long whistles were in a few cases misclassified as Short whistles. Trills were correctly classified in all cases (Supplementary Table S4). The recall was high for all call types (Long whistles: median = 0.80, IQR = 0.23, Trill: median = 1.00, IQR = 0.00, Short whistles: median = 0.91, IQR = 0.36, Tsak: median = 0.98, IQR = 0.14, Zip: median: 1.00, IQR = 0.00; Fig. 6b).

### Unsupervised clustering as a tool to automate the establishing of call categories (Model 3)

**Figure 7:**
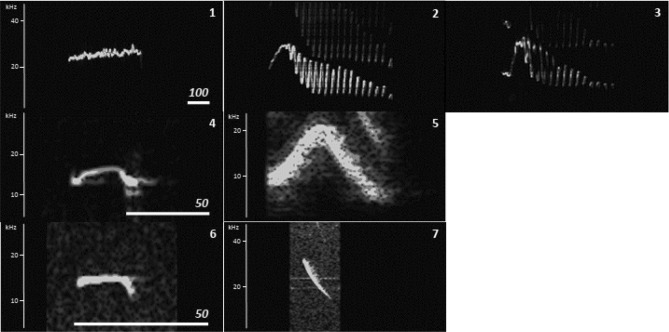
Top categories identified by the clustering model. The white bars depict the time scale in milliseconds.

The detected calls from *M. murinus* audio recordings were clustered by the k-means model into seven different clusters (Fig. 7, Supplementary Fig. S5). Cluster 1 matched the Long whistles predefined category; cluster 2 and 3, Trills; cluster 4 partly matched Tsaks and Short whistles; cluster 5, Tsaks; cluster 6, Short whistles; and cluster 7 matched predefined Zip calls. Thus, according to the model, Long whistles and Zips had one cluster each; whereas Trills had two clusters (Fig. 7). In addition, the model grouped part of the predefined Short whistle and Tsak calls into one common cluster.

## Discussion

This study shows that DeepSqueak can successfully detect primate vocalizations in the high-frequency and ultrasonic domain under different quality scenarios at least under laboratory conditions. Furthermore, automated labeling of the data set and clustering can follow up the detection process with minimal to no human intervention without running into considerable performance loss. Unwanted noise signals and fragments could be removed by the DS-Denoiser and R-DS Filter, respectively. The major drawback is the clustering of calls by the Short and Very short detectors, which can be fixed by a semi-automated approach. The unsupervised clustering distinguished more than five clusters. However, merging some of the clusters predicted by the model would result in categories representing the five human-made call types. Overall, DeepSqueak offers a user-friendly automated analysis for primate vocalizations and, thereby can be used as a cross-taxa tool to speed up animal communication analyses, and reduce subjectivity for the steps of detection, categorization and classification of vocalizations.

### Effect of individual variation, recording quality and species on detection performance (Model 1)

When using automated detection, DeepSqueak detected 91% of the gray mouse lemur vocalizations from the experimental data set recorded under laboratory conditions with high precision (median detection recall = 0.91, median detection precision = 0.90). This performance is comparable to other studies (rodents^25^: recall > 0.80, precision > 0.9; marmoset monkeys^26^: recall: 0.77–0.85; precision: 0.68–0.79). Thus, the sample of the training data was sufficient to achieve a good performance of the detector networks. Thereby, the three trained detector networks worked as intended: Long whistles and Trills were best detected by the Long detector, Short whistles and Tsaks by the Short detector. The Zips were detected by both, the Short and Very short detector. However, even if the Zips were detected by the Short detector using the standardized data set, using the experimental data set Zips were only detected by the Very short detector. Thus, bringing chunks from different audio recordings together into one file may produce an artifact that made the two Short detectors undistinguishable from each other. Such an artifact might result from different background amplitude levels of the audio chunks, hampering the DeepSqueak’s filtering ability to set a general reference boundary condition to differentiate between signal and noise. This has to be taken into account when merging different audio recordings or single calls.

We further showed that the detectors are robust against both intraspecific variation in call structure and different recording quality scenarios. The results of the good-quality standardized data sets showed that the detector networks detected the calls even in the presence of intraspecific variation. This is important for studies on animal vocalizations when calls are naturally plastic, as is the case for the mouse lemur species. Animals usually modulate, to a certain extent, some components of their calls, which can be due to genetic, behavioral/motivational, physiological, allometric and environmental reasons (e.g., Refs.^43,47,48,54,60,61,68–71^). In the presence of such modulations, over-fitted networks may often miss opportunities (i.e., increased false negatives) and yield low recall, even for good-quality recordings. Thus, the use of diverse training data—as done in this study—helps to obtain detector networks, which are generally sufficiently robust to perform well in the presence of intraspecific variation.

In addition, all three detectors proved robust in a large range of audio quality conditions typical for animal recordings (clipped, low-amplitude, overlaid calls). Clipped calls were typically well detected (i.e., relatively high precision and recall), though many of the short calls were clustered. When the signals were of low-amplitude, the precision was still high, meaning that detections mainly hit the target sounds. However, low-amplitude signals were often overlooked by the detectors, yielding a relatively low recall. This can be expected, since amplitude variations in low-amplitude calls are barely different from the background noise of the recording. Overlaid calls were detected, clustering always with the overlapping calls. Thus, overlaid calls can still be counted but not measured, even when not using a manual scanning approach.

The performance of the detectors was comparable between the target species (*M. murinus*) and an evolutionary closely related species (*M. lehilahytsara* Fig. 5c,d). This shows that the trained detectors can be used for detecting the vocalizations of other mouse lemur species, at least as starting points.

### Recommendations for the use of DeepSqueak for experimental data (Model 1)

Signal recognition (detection task) is one of the most challenging steps in the process of automated vocalization analysis, since a high recall in the process of signal recognition (i.e., detection process) is accompanied by a drop in precision due to the presence of unwanted noise sources (e.g., researcher speaking, nearby machines, movement of animals, their interactions or other sources). In this study, the precision was still relatively high, even in the presence of unwanted noise and call fragments (automated approach), which is comparable to other studies (rodents^25^: precision > 0.9; marmoset monkeys^26^: maximum precision: 0.79). This was achieved by the implementation of two filtering processes: (1) a classification network removing unwanted noise (named DS-Denoiser), and (2) a custom R script fixing call fragments (the R-DS Filter).

The coupling of machine learning models (detectors + DS-Denoiser) has been useful to reduce the amount of false positives from audio recordings with high signal heterogeneity, typical for recordings in the wild^72^. Thus, whenever unwanted noise is expected or identified during visual inspection of detection files, the use of a denoiser is recommended.

Using all detectors simultaneously (multi-detector), the Short and the Very short detectors detect call fragments of long calls as hits due to amplitude variation in the whole call. Such unwanted fragments cannot be used for measuring the whole call and would falsify call rates. Thus, we recommend using the R-DS Filter to merge call fragments and thereby considerably reduce misdetections (but if the researcher is interested in extracting single syllables of the Trill, the Short and Very short detectors can be used to extract these syllables). In addition, combining DeepSqueak with R has the advantage that the user can have more control over the measurement of relevant features from the calls by implementing additional analyses (e.g., when measuring syllables in R with PRAAT^73^).

Though a good performance was achieved using the automated approach, the performance could be remarkably improved by visual inspection of the detection files using the GUI in DeepSqueak (semi-automated approach) and thus proceed to edit the measuring box, reject/accept calls, if necessary. For instance, often in this study, short calls were detected as clusters instead of single call elements. This was observed when using the Short detector on the standardized data set, and, exceptionally, when using all three detectors simultaneously on the experimental data set. Whenever calls are detected in clusters, manual editing of the measuring boxes using the GUI can help to detect each call independently. Thus, we recommend conducting a visual scanning of the detection files and edit the measuring boxes, if necessary (semi-automated approach). This is especially important when scientists are interested in call rates. However, if researchers are interested in specific call types, we recommend using the corresponding detector network based on the call duration. Using the semi-automatic approach, recall values sometimes exceed one, demonstrating that the detector detected more target calls than were detected by manual screening of bioacoustic experts. This was especially true for audio files which contained few low-amplitude short calls (duration up to 30 ms), that could be easily overseen from long audio files (in this study, audio files mean duration: 14 min) or several hundred vocalizations combined to a series (Short whistle and Tsak). This shows that even if the detector can make errors, the human observer makes errors as well, emphasizing the problem of reliably screening audio files, which contain thousands of vocalizations and/or are of long duration.

### Supervised classification as a tool to automatically label call types (Model 2)

The supervised classifier network trained using DeepSqueak built-in routines had a good performance for most call types (median precision = 1.00; median recall > 0.80 for all call types except Zips) comparable to other studies (lemur^27^: precision > 0.90, marmosets^31^: median precision 0.84, median recall 0.81). Only for Zips the precision was very low (median precision = 0.14). This could be either explained by the rare occurrence of Zips combined with a high call rate of Short whistles, which were sometimes misclassified as Zips. For example, an audio file with two Zip calls (*tt* = *2*) and 709 Short whistles had 12 misclassifications. All 12 misclassification were Short whistles misclassified as Zips, resulting in a precision of 0.14 for Zips. However, all true Zips were correctly classified in all cases, resulting in a recall of 1.0. Thus, in such cases where one call type occurred very rarely, we recommend using the semi-automated approach, which can considerably reduce the amount of false positives during the classification task (personal observation) due to manual adjustment of measuring boxes.

### Unsupervised clustering as a tool to “objectively” categorize call types (Model 3)

The clustering model identified seven clusters (Supplementary Fig. S5), of which two matched the predefined Trills, and one consisted of predefined Short whistles and some Tsak calls. Thus, the resulting categories were fairly similar to human-made categories^49,50^. The small discrepancies between the clustering method and human-made categories can be expected, given the multi-dimensional space on which the algorithm based its decisions, which may not always match the features used by human observers. Moreover, transitions between different call types can be observed (e.g., Short whistle to Tsak, Short whistle to Long whistle). Thus, it is not surprising that clusters occurred containing Short whistles and Long whistles or Tsaks. Such transitions can make a manual human-made approach less reliable and dependent on the experimenter’s experience. The unsupervised clustering model can help reduce this subjectivity^28^, and can potentially increase reproducibility of results across studies. This does not mean that the AI will be perfect. Rather, it means that it will at least be consistent with the “mistakes” it makes. Furthermore, the clustering models in DeepSqueak are based solely on acoustic parameters and therefore can result in arbitrary clusters which are not related to biologically relevant information. Therefore, it is recommended to evaluate not only the consistency of the results from the clustering models but also to relate them to the animals behavior and the auditory skills (e.g., temporal and frequency resolution of the auditory system).

Conclusively, this study shows that DeepSqueak, initially developed for detection and analysis of mouse and rat ultrasonic vocalizations, can be successfully utilized to detect vocalizations of other taxa in the high-frequency to ultrasonic range with high precision and recall. The performance was robust against individual variation, and when using the semi-automated approach it can deal with different recording qualities. Unwanted noise signals and call fragments, typical problems of these kinds of approaches, were drastically reduced through the use of the DS-Denoiser (a classification network trained in DeepSqueak) and R-DS Filter developed in R. Thereby, the detector networks could also be used to detect vocalizations of an evolutionary closely related species. Thus, DeepSqueak seems to be a user-friendly tool, which can be utilized for different mammalian species. Its dependence on MATLAB (The MathWorks, Inc., Natick, MA, USA) as an expensive source platform might be still a disadvantage, but we hope that our results can inspire future open-source end-to-end approaches using faster regional convolutional neural networks.

## Supplementary Information

Supplementary Information 1.

Supplementary Information 2.

## Data Availability

The data, detector and classification networks, the DS-Denoiser network and filtering R-DS routine, and the clustering model used in this study are available in a GitHub repository (https://github.com/M0rph3u2x/How-Deepsqueak-can-be-utilized-for-mammalian-vocalizations).

## References

[1] Priyadarshani N, Marsland S, Castro I (2018). Automated birdsong recognition in complex acoustic environments: A review. J. Avian Biol..

[2] Barker DJ, Johnson AM (2017). Automated acoustic analysis of 50-kHz ultrasonic vocalizations using template matching and contour analysis. J. Acoust. Soc. Am..

[3] Oswald JN, Rankin S, Barlow J, Lammers MO (2007). A tool for real-time acoustic species identification of delphinid whistles. J. Acoust. Soc. Am..

[4] Van Segbroeck M, Knoll AT, Levitt P, Narayanan S (2017). MUPET—Mouse Ultrasonic Profile ExTraction: A signal processing tool for rapid and unsupervised analysis of ultrasonic vocalizations. Neuron.

[5] Binder MS, Hernandez-Zegada CJ, Potter CT, Nolan SO, Lugo JN (2018). A comparison of the Avisoft (5.2) and Ultravox (2.0) recording systems: Implications for early-life communication and vocalization research. J. Neurosci. Methods.

[6] Mcloughlin MP, Stewart R, McElligott AG (2019). Automated bioacoustics: Methods in ecology and conservation and their potential for animal welfare monitoring. J. R. Soc. Interface.

[7] Castellote M, Fossa F (2006). Measuring acoustic activity as a method to evaluate welfare in captive beluga whales (*Delphinapterus leucas*). Aquat. Mamm..

[8] Clapham WM, Fedders JM, Beeman K, Neel JPS (2011). Acoustic monitoring system to quantify ingestive behavior of free-grazing cattle. Comput. Electron. Agric..

[9] Schön PC (2007). Altered vocalization rate during the estrous cycle in dairy cattle. J. Dairy Sci..

[10] Cascão I, Lammers MO, Prieto R, Santos RS, Silva MA (2020). Temporal patterns in acoustic presence and foraging activity of oceanic dolphins at seamounts in the Azores. Sci. Rep..

[11] Manteuffel GR, Schön PC (2004). STREMODO, an innovative technique for continuous stress assessment of pigs in housing and transport. Arch. Tierzucht..

[12] Chedad A (2001). Recognition system for pig cough based on probabilistic neural networks. J. Agric. Eng. Res..

[13] Bardeli R (2010). Detecting bird sounds in a complex acoustic environment and application to bioacoustic monitoring. Pattern Recogn. Lett..

[14] Jones, K. E. *et al.* In *Biodiversity Monitoring and Conservation: Bridging the Gap Between Global Commitment and Local Action* (eds Collen, B., *et al*.) Ch. 10, (Taylor & Francis, 2013).

[15] Marques TA (2013). Estimating animal population density using passive acoustics. Biol. Rev..

[16] Stevenson BC (2015). A general framework for animal density estimation from acoustic detections across a fixed microphone array. Methods Ecol. Evol..

[17] Wrege PH, Rowland ED, Keen S, Shiu Y (2017). Acoustic monitoring for conservation in tropical forests: Examples from forest elephants. Methods Ecol. Evol..

[18] Haver SM (2019). Comparing the underwater soundscapes of four U.S. national parks and marine sanctuaries. Front. Mar. Sci..

[19] Beason RD, Riesch R, Koricheva J (2019). AURITA: An affordable, autonomous recording device for acoustic monitoring of audible and ultrasonic frequencies. Bioacoustics.

[20] Beeman KH, Hopp SL, Owren MJ, Evans CSE (1998). Animal Acoustic Communication: Sound Analysis and Research Methods.

[21] Janik VM (1999). Pitfalls in the categorization of behaviour: A comparison of dolphin whistle classification methods. Anim. Behav..

[22] Gillespie D (2009). PAMGUARD: Semiautomated, open source software for real-time acoustic detection and localization of cetaceans. J. Acoust. Soc. Am..

[23] Kaleidoscope Pro Analysis Software [Software]. (Wildlife Acoustics, Inc. https://www.wildlifeacoustics.com (2020).

[24] Ruff ZJ, Lesmeister DB, Duchac LS, Padmaraju BK, Sullivan CM (2020). Automated identification of avian vocalizations with deep convolutional neural networks. Remote Sens. Ecol. Conserv..

[25] Coffey KR, Marx RG, Neumaier JF (2019). DeepSqueak: A deep learning-based system for detection and analysis of ultrasonic vocalizations. Neuropsychopharmacology.

[26] Oikarinen T (2019). Deep convolutional network for animal sound classification and source attribution using dual audio recordings. J. Acoust. Soc. Am..

[27] Pozzi L, Gamba M, Giacoma C (2010). The use of artificial neural networks to classify primate vocalizations: A pilot study on black lemurs. Am. J. Primatol..

[28] Gamba M (2015). Comparative analysis of the vocal repertoire of *Eulemur*: A dynamic time warping approach. Int. J. Primatol..

[29] Pozzi, L., Gamba, M. & Giacoma, C. In *Leaping Ahead: Advances in Prosimian Biology.* (ed Masters, J.) Ch. 34, 305–313 (Springer, 2013).

[30] Heinicke S (2015). Assessing the performance of a semi-automated acoustic monitoring system for primates. Methods Ecol. Evol..

[31] Turesson HK, Ribeiro S, Pereira DR, Papa JP, de Albuquerque VHC (2016). Machine learning algorithms for automatic classification of marmoset vocalizations. PLoS One.

[32] Bergler C (2019). ORCA-SPOT: An automatic killer whale sound detection toolkit using deep learning. Sci. Rep..

[33] Shiu Y (2020). Deep neural networks for automated detection of marine mammal species. Sci. Rep..

[34] Zeppelzauer M, Hensman S, Stoeger AS (2015). Towards an automated acoustic detection system for free-ranging elephants. Bioacoustics.

[35] Venter PJ, Hanekom JJ (2010). Automatic detection of African elephant (*Loxodonta africana*) infrasonic vocalisations from recordings. Biosyst. Eng..

[36] Mac Aodha O (2018). Bat detective-Deep learning tools for bat acoustic signal detection. PLoS Comput. Biol..

[37] Henriquez A (2014). An automatic acoustic bat identification system based on the audible spectrum. Expert Syst. Appl..

[38] Hoy MB (2018). Alexa, Siri, Cortana, and more: An introduction to voice assistants. Med. Ref. Serv. Q..

[39] López, G., Quesada, L. & Guerrero, L. A. In *Advances in Human Factors and Systems Interaction. AHFE 2017. Advances in Intelligent Systems and Computing* Vol. 592 (ed. Nunes, I.) (Springer, 2018).

[40] Ren S, He K, Girshick R, Sun J (2017). Faster R-CNN: Towards real-time object detection with region proposal networks. IEEE Trans. Pattern Anal. Mach. Intell..

[41] Barker DJ, Herrera C, West MO (2014). Automated detection of 50-kHz ultrasonic vocalizations using template matching in XBAT. J. Neurosci. Methods.

[42] Zimmermann, E. In *Leaping Ahead: Advances in Prosimian Biology* (eds. Masters, J., Gamba, M., & Génin, F.) Ch. 32, 287–295 (Springer, 2013).

[43] Schopf C, Schmidt S, Zimmermann E (2016). Moderate evidence for a Lombard effect in a phylogenetically basal primate. PeerJ.

[44] Niaussat MM, Petter JJ (1980). Etude de la sensibilité auditive d'un lémurien malgache: *Microcebus murinus* (J.-F. Miller, 1777). Mammalia.

[45] Hasiniaina AF (2020). Evolutionary significance of the variation in acoustic communication of a cryptic nocturnal primate radiation (*Microcebus* spp.). Ecol. Evol..

[46] Braune P, Schmidt S, Zimmermann E (2008). Acoustic divergence in the communication of cryptic species of nocturnal primates (*Microcebus* ssp.). BMC Biol..

[47] Leliveld LMC, Scheumann M, Zimmermann E (2011). Acoustic correlates of individuality in the vocal repertoire of a nocturnal primate (*Microcebus murinus*). J. Acoust. Soc. Am..

[48] Scheumann M, Zimmermann E, Deichsel G (2007). Context-specific calls signal infants' needs in a strepsirrhine primate, the gray mouse lemur (*Microcebus murinus*). Dev. Psychobiol..

[49] Zimmermann, E. In *Handbook of Mammalian Vocalization: An Integrative Neuroscience Approach.* (ed. Brudzynski, S. M.) 215–225 (Academic Press, 2010).

[50] Zimmermann, E. In *Handbook of Ultrasonic Vocalization: A Window into the Emotional Brain* vol. 25 (ed. Brudzynski, S. M.) 521–533 (Academic Press, 2018).

[51] Buesching CD, Heistermann M, Hodges JK, Zimmermann E (1998). Multimodal oestrus advertisement in a small nocturnal prosimian, *Microcebus murinus*. Folia Primatol..

[52] Scheumann M, Linn S, Zimmermann E (2017). Vocal greeting during mother–infant reunions in a nocturnal primate, the gray mouse lemur (*Microcebus murinus*). Sci. Rep..

[53] Braune P, Schmidt S, Zimmermann E (2005). Spacing and group coordination in a nocturnal primate, the golden brown mouse lemur (*Microcebus ravelobensis*): The role of olfactory and acoustic signals. Behav. Ecol. Sociobiol..

[54] Kessler SE, Scheumann M, Nash LT, Zimmermann E (2012). Paternal kin recognition in the high frequency/ultrasonic range in a solitary foraging mammal. BMC Ecol..

[55] Zimmermann E, Hafen TG (2001). Colony specificity in a social call of mouse lemurs (*Microcebus* ssp.). Am. J. Primatol..

[56] Hafen T, Neveu H, Rumpler Y, Wilden I, Zimmermann E (1998). Acoustically dimorphic advertisement calls separate morphologically and genetically homogenous populations of the grey mouse lemur (*Microcebus murinus*). Folia Primatol..

[57] Zimmermann E, Lerch C (1993). The complex acoustic design of an advertisement call in male mouse lemurs (*Microcebus murinus*, Prosimii, Primates) and sources of its variation. Ethology.

[58] Zimmermann E (1996). Castration affects the emission of an ultrasonic vocalization in a nocturnal primate, the grey mouse lemur (*Microcebus murinus*). Physiol. Behav..

[59] Keenan S, Lemasson A, Zuberbühler K (2013). Graded or discrete? A quantitative analysis of Campbell's monkey alarm calls. Anim. Behav..

[60] Tallet C (2013). Encoding of situations in the vocal repertoire of piglets (*Sus scrofa*): A comparison of discrete and graded classifications. PLoS One.

[61] Hasiniaina AF (2018). High frequency/ultrasonic communication in a critically endangered nocturnal primate, Claire's mouse lemur (*Microcebus mamiratra*). Am. J. Primatol..

[62] Boersma P (2001). Praat, a system for doing phonetics by computer. Glot Int..

[63] Owren MJ (2008). GSU Praat Tools: Scripts for modifying and analyzing sounds using Praat acoustics software. Behav. Res. Methods.

[64] *R: A Language and Environment for Statistical Computing* (R Foundation for Statistical Computing, 2020).

[65] Fränti P, Sieranoja S (2019). How much can k-means be improved by using better initialization and repeats?. Pattern Recogn..

[66] Patterson, J. & Gibson, A. *Deep Learning: A Practitioner’s Approach*. (O’Reilly Media, Inc., 2017).

[67] Field, A. *Discovering Statistics Using IBM SPSS Statistics (Englisch)*. 3rd ed. (Sage Publication, 2009).

[68] Clink DJ, Tasirin JS, Klinck H (2019). Vocal individuality and rhythm in male and female duet contributions of a nonhuman primate. Curr. Zool..

[69] Romero-Mujalli D, Tárano Z, Cobarrubia S, Barreto G (2014). Caracterización de silbidos de Tursiops truncatus (Cetacea: Delphinidae) y su asociación con el comportamiento en superficie. Revista Argentina de Ciencias del Comportamiento.

[70] Papale E, Gamba M, Perez-Gil M, Martin VM, Giacoma C (2015). Dolphins adjust species-specific frequency parameters to compensate for increasing background noise. PLoS One.

[71] García NC, Barreira AS, Kopuchian C, Tubaro PL (2014). Intraspecific and interspecific vocal variation in three *Neotropical cardinalids* (Passeriformes: Fringillidae) and its relationship with body mass. Emu.

[72] Lostanlen V, Salamon J, Farnsworth A, Kelling S, Bello JP (2019). Robust sound event detection in bioacoustic sensor networks. PLoS One.

[73] Albin A (2014). PraatR: An architecture for controlling the phonetics software “Praat” with the R programming language. J. Acoust. Soc. Am..

