# Supplementary material for: Utilizing DeepSqueak for automatic detection and classification of mammalian vocalizations: a case study on primate vocalizations
Source: Sci Rep. 2021 Dec 27;11:24463. doi: 10.1038/s41598-021-03941-1 (PMC8712519; doi:10.1038/s41598-021-03941-1)
Supplement: Supplementary file 1 — Supplementary Information 1. [file 41598_2021_3941_MOESM1_ESM.docx]

**Utilizing DeepSqueak for automatic detection and classification of mammalian vocalizations: A case study on primate vocalizations**

Daniel Romero-Mujalli^1*^, Tjard Bergmann^1^, Axel Zimmermann^2^, Marina Scheumann^1^

^1^ Institute of Zoology, University of Veterinary Medicine Hannover, Hannover, Germany

^2^ University of Aalen, Aalen, Germany

*Corresponding author: Daniel Romero-Mujalli^1^

University of Veterinary Medicine Hanover

Institute of Zoology

Bünteweg 17

30559 Hannover

Germany

daniel.romero.mujalli@tiho-hannover.de

**Supplementary Information**

**Supplementary Table S1:** Overview about the audio recordings used in this study: Experimental conditions and recording equipment. Social encounter paradigm: Two subjects (same sex or opposite sex) were positioned in two test cages, which were connected by a door. Animals were observed for 30 minutes with the door closed and 30 minutes with an open door. This paradigm evoked all call types. Handling of animals: A subject was recorded while opening the nest box, taking the animal out and holding the animal in the experimenter’s hand. The paradigm evoked Tsaks and Short whistles (also Grunt and Croaks). Mother-infant reunion: An infant was placed in a test cage and the mother was released. The experiment lasted until the mother had carried the baby back to the nest or for a maximum of 10 minutes. This paradigm evoked Trills and Short whistles of the mother. Playback: A subject was placed in a test cage and calls from conspecifics, heterospecifics, predators or infants were played back from a loudspeaker. MFR = microphone frequency response.

| Data set | Experimental paradigm | Recording equipment |
| --- | --- | --- |
| Training  and standardized data set | Social encounters^1,2,3^,  Handling of animals^1^,  Mother-infant reunions^1,2,3^,  Playback of predators^3^,  Playback of infant calls^1,2,3^,  Playback of conspecific and heterospecific calls^3^ | ^1^Pettersson D1000X ultrasound detector (16-bit, 250 kHz sampling rate); MFR: 5-235 kHz |
|  |  | ^2^U30 Batdetector via analog/digital filter (DAQCard-6062E) linked to a computer (12-bit, 200-500 kHz sampling rate); MFR: 8–100 kHz |
|  |  | ^3^U30 Batdetector via control-filter unit (Pettersonbox F2000) linkted to an analog/digital converter (PCM-DAS 16/330) in a laptop (12-bit, 200 kHz); MFR: 8–100 kHz |
| Experimental data set | Female-male social encounters | *M. murinus*^43^:  Pettersson D1000X ultrasound detector (16-bit, 250 kHz sampling rate); MFR: 5-235 kHz  *M. lehilahytsara:*  U30 Batdetector via analog/digital filter (DAQCard-6062E) linked to a computer (12-bit, 200 kHz sampling rate); MFR: 8-100 kHz |

**Supplementary Table S2:** Definition of automatically extracted acoustic parameters based on the contour detection. Using the contour detection method for each time point, the maximum amplitude was detected and then the spectrogram was cleaned by removing non-tonal features, based on the concept of tonality (for more details see the Wiki of DeepSqueak^25^ <https://github.com/DrCoffey/DeepSqueak/wiki/export-to-excel>, 3.06.2021).

| **Acoustic parameter** | **Description** |
| --- | --- |
| **Duration [ms]** | Time from the start to the end of contour detection |
| **Principal freq.(kHz)** | Mean frequency of the contour |
| **Min. freq. (kHz)** | Minimum frequency of the contour |
| **Max. freq. (kHz)** | Maximum frequency of the contour |
| **Bandwidth (kHz)** | Maximum minus minimum frequency of the contour |
| **Slope (kHz / s)** | Slope of the contour |
| **Sinuosity** | Length of the contour between the first and last points divided by the euclidean distance between the first and last points |
| **Mean power (db)** | Mean power spectral density of the contour |
| **Tonality** | One minus the Wiener entropy measured by dividing the geometric mean by the arithmetic mean |

**Supplementary Table S3:** Acoustic description of the five tonal call types investigated in this study (mean ± standard deviation); Long whistle - LW, Short whistle - SW, Trill - TR, Tsak - TS, Zip - ZP. The measurements are based on the automated detections using the best detector (Long detector for LW and TR; Short detector for SW, TS and ZP) and if necessary the measurement boxes were manually adapted to the contour of the fundamental frequency. Afterwards, all parameters were automatically extracted by DeepSqueak based on the contour detection method.

| **Call type** | **Duration (ms)** | **Principal freq. (kHz)** | **Min. freq. (kHz)** | **Max. freq. (kHz)** | **Bandwidth (kHz)** | **Slope (kHz / s)** | **Sinuosity** | **Mean power (db)** | **Tonality** |
| --- | --- | --- | --- | --- | --- | --- | --- | --- | --- |
| LW (N=46) | 388.5 ± 172.64 | 19.85 ± 3.48 | 18.23 ± 3.44 | 20.87 ± 3.33 | 2.63 ± 1.44 | 1.44 ± 5.09 | 1.22 ± 0.33 | -52.72 ± 7.05 | 0.87 ± 0.05 |
| SW  (N=86) | 33.94 ± 10.99 | 14.65 ± 2.39 | 13.26 ± 2.51 | 15.1 ± 2.36 | 1.84 ± 1.38 | -1.23 ± 28.31 | 1.07 ± 0.08 | -64.07 ± 7.15 | 0.7 ± 0.07 |
| TR  (N=40) | 646.79 ± 138.57 | 21.19 ± 3.76 | 11.50 ± 3.86 | 33.35 ± 6.05 | 21.89 ± 7.37 | -17.33 ± 7.13 | 12.96 ± 5.97 | -62.45 ± 6.17 | 0.61 ± 0.11 |
| TS  (134) | 52.03 ± 12.66 | 12.4 ± 1.97 | 8.39 ± 1.75 | 17.69 ± 3.83 | 9.29 ± 4.54 | -65.54 ± 74.96 | 1.58 ± 0.53 | -63.47 ± 6.08 | 0.63 ± 0.07 |
| ZP  (N=50) | 12.45 ± 4.22 | 26.3 ± 2.17 | 21.14 ± 2.51 | 31.83 ± 3.2 | 10.68 ± 3.65 | -943.56 ± 237.17 | 1.09 ± 0.25 | -62.61 ± 3.45 | 0.5 ± 0.06 |

**Supplementary Table S4:** Confusion matrix considering the classification results over all examined audio recordings. LW = Long whistle; TR = Trill; SW = Short whistle; TS =Tsak; ZP = Zip; N_total true_ = total number of calls in the audio files; N_correct_ = total number of calls which were predicted by the model; false negative = total number of calls in the audio files, which were not predicted by the model to the correct call type; False positive = total number of calls, which were wrongly classified to the respective call type; bold numbers = correct predictions; non-bold numbers = wrong predictions.

|  |  | **Predicted call type** | | | | | **N_total true_** | **N_correct_** | **False negative** | **False positive** |
| --- | --- | --- | --- | --- | --- | --- | --- | --- | --- | --- |
|  |  | **LW** | **TR** | **SW** | **TS** | **ZP** |  |  |  |  |
| **True call type** | **LW** | **111** | 3 | 23 | 0 | 1 | 138 | **111** | 27 | 16 |
|  | **TR** | 0 | **73** | 0 | 0 | 0 | 73 | **73** | 0 | 5 |
|  | **SW** | 16 | 1 | **2256** | 11 | 119 | 2403 | **2256** | 147 | 24 |
|  | **TS** | 0 | 1 | 1 | **42** | 2 | 46 | **42** | 4 | 11 |
|  | **ZP** | 0 | 0 | 0 | 0 | **23** | 23 | **23** | 0 | 122 |
|  | **Total** |  |  |  |  |  | 2683 | **2505** | 178 | 178 |


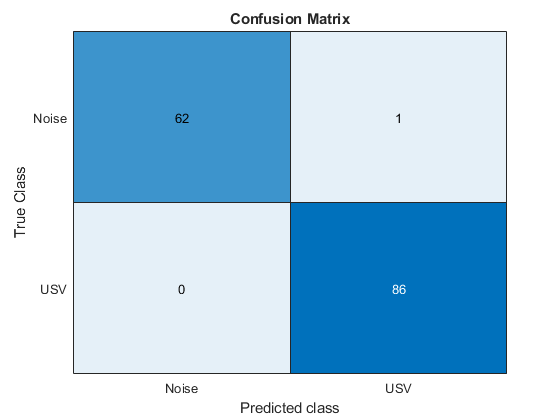


**Figure S1:** Confusion matrix after training the denoiser network on the corresponding training data using the DeepSqueak built-in training routine.


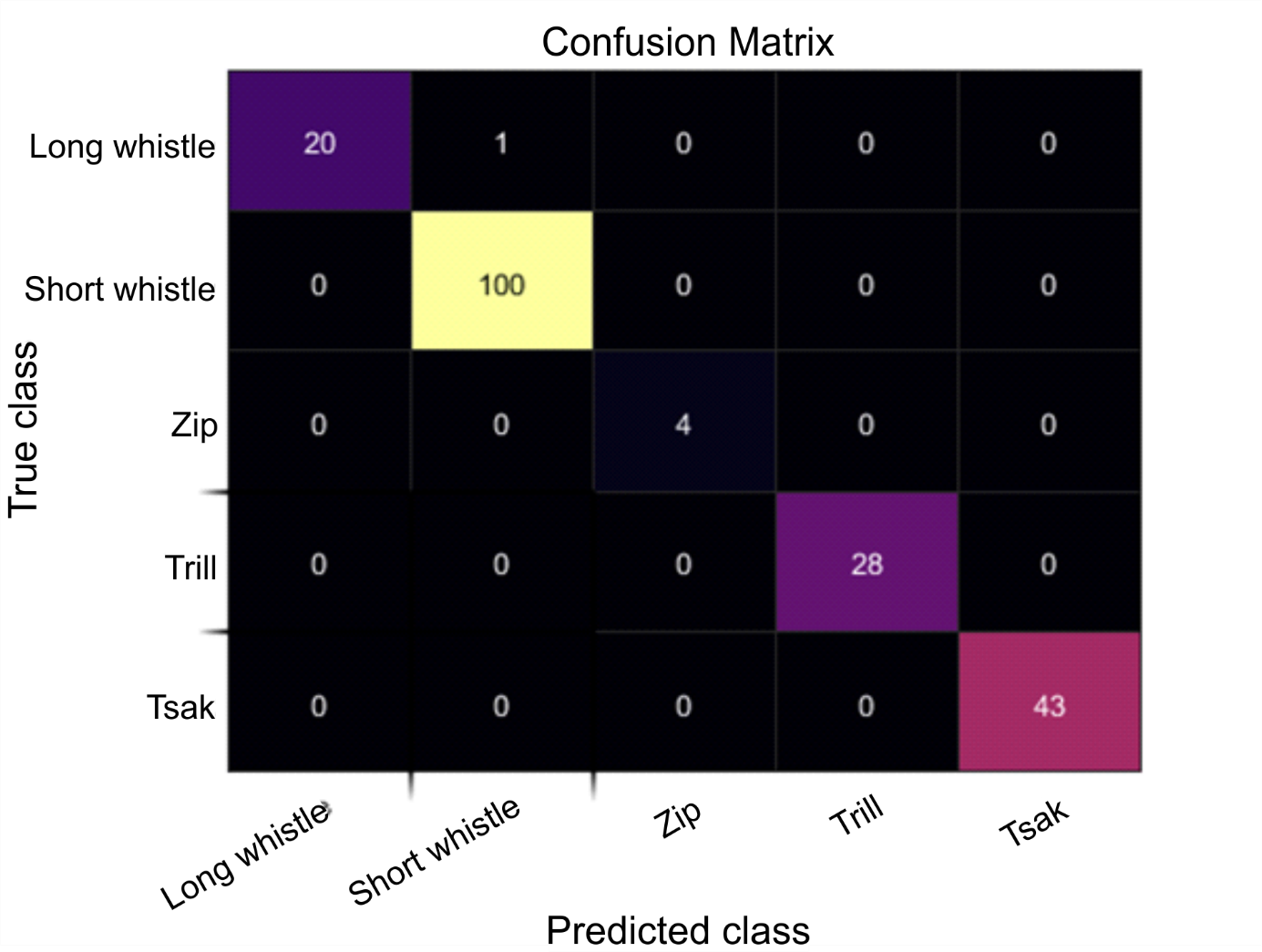


**Figure S2:** Confusion matrix after the training of the classifier network using the corresponding DeepSqueak built-in training routine.


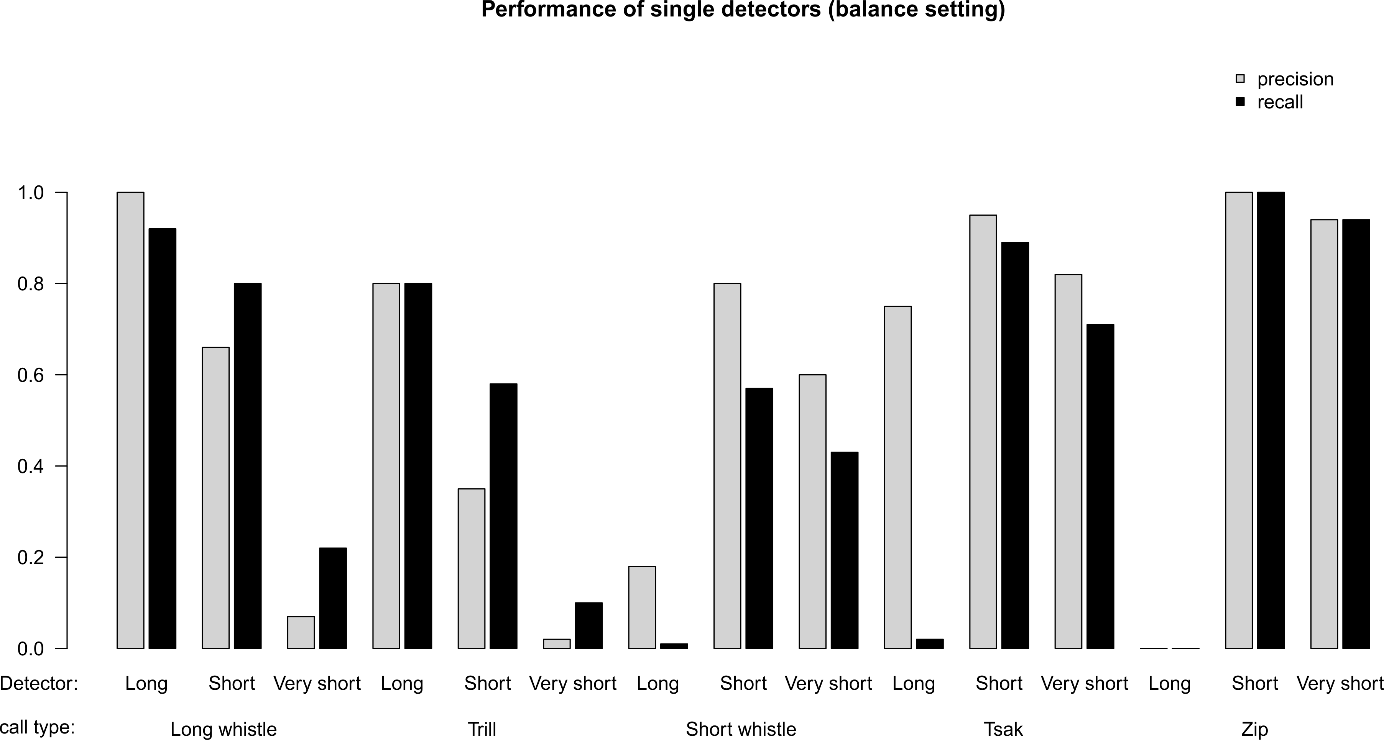


**Figure S3:** Bar plots of the performance (precision, gray; recall, black) of single detector networks (Long, Short, Very short detectors) per call types (long calls: Long whistle and Trill; short calls: Short whistle and Tsak; very short calls: Zip) tested on the good-quality standardized data sets (one call type per file).

**
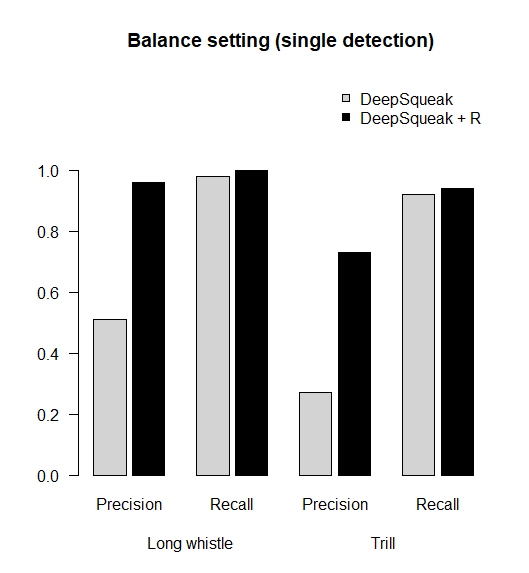
**

**Figure S4:** Bar plots of the precision before and after applying the R-DS Filter to correct for the call fragments of Long whistles and Trills being identified by the Short detectors when using multiple detectors simultaneously.


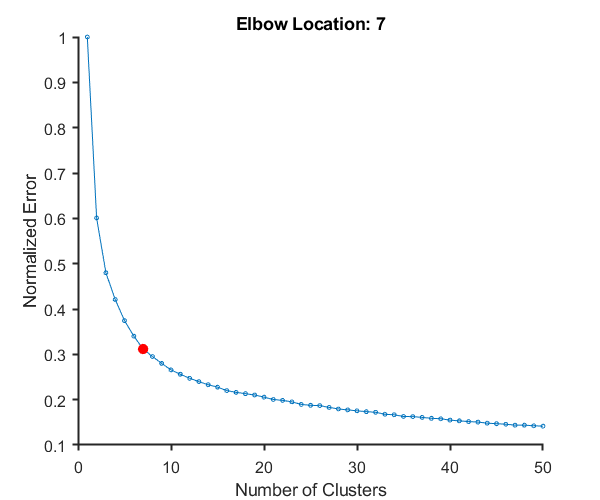


**Figure S5:** Plot showing the optimum number of clusters based on the Elbow method^25^ (unsupervised clustering, K-means model).
