# Supplementary material for: Utilizing DeepSqueak for automatic detection and classification of mammalian vocalizations: a case study on primate vocalizations
Source: Sci Rep. 2021 Dec 27;11:24463. doi: 10.1038/s41598-021-03941-1 (PMC8712519; doi:10.1038/s41598-021-03941-1)
Supplement: Supplementary file 2 — Supplementary Information 2. [file 41598_2021_3941_MOESM2_ESM.docx]

**Utilizing DeepSqueak for automatic detection and classification of mammalian vocalizations: A case study on primate vocalizations**

Daniel Romero-Mujalli^1*^, Tjard Bergmann^1^, Axel Zimmermann^2^, Marina Scheumann^1^

^1^ Institute of Zoology, University of Veterinary Medicine Hannover, Hannover, Germany

^2^ University of Aalen, Aalen, Germany

*Corresponding author: Daniel Romero-Mujalli

University of Veterinary Medicine Hanover

Institute of Zoology

Bünteweg 17

30559 Hannover

Germany

danielrm84@gmail.com

**Supplementary Methods**

**Additional information on the preparation of the standardized data sets**

*Standardized data set*

The preprocessing and combination were performed with the software PRAAT (www.praat.org)^62^ using the GSUPraatTool 1.9 ^63^. The standardized data set consisted of single or series of vocalizations combined into an audio file. Before concatenating the single vocalizations, we preprocessed the vocalization. We removed direct current (DC) offsets and, if necessary, resampled the files with the same sample frequency of 200 kHz to guarantee the same frequency resolution for all files used in this study using the “preprocess” function of the GSUPraatTool. Afterwards, we combined the single calls/series of each call type to an audio file using the “concatenate” function of PRAAT. We added between each call/series an interval of three seconds of white noise with an amplitude intensity of 28 db to simulate real case audio files where calls are separated by background noise.

We created standardized data sets of four different recording quality scenarios. The scenarios were established manually by bioacoustics experts (MS) using the following definitions:

*Good quality*: Calls with a signal to noise ratio above 5%, not clipped, nor overlapped with other call types and/or disturbance bands in the frequency range of 5-50 kHz. For Long whistle, Trill and Zip files of 50 single calls and for Tsaks and Short whistles of 50 series (150 single calls) were used. Vocalizations originated from different individuals to test the robustness of the detector against potential individual variation.

*Clipped:* Calls which were overloaded, not overlaid and without disturbance bands in the frequency range of 5-50 kHz. For each scenario, ten calls/series per call type were used.

*Low-amplitude:* Call with a signal to noise ratio below 5 %, not clipped, not overlaid with other call types and/or disturbance bands in the frequency range of 5-50 kHz.

*Overlaid:* Calls where at the same time, another call type from a social partner

was uttered, not clipped and without disturbance bands in the frequency

range of 5-50 kHz.

**Additional information on the setting of the training**

DeepSqueak created sonogram images of the selected vocalizations using a non-uniform fast fourier transformation with different settings depending on the target's length (long, short or very short calls) (setting: Long whistle and Trill: Window = 0.01 s, Overlap = 0.005 s, NFFT = 0.01 s; Short whistle, Tsak and Zip: Window = 0.0032 s, Overlap = 0.0028 s and NFFT = 0.0032 s). Two training images were created per selected vocalization (duplication parameter was set to one). Only for the Zips, was the duplication parameter set to 30 as such that the Zips training examples were sufficiently large. Thus, a total of 4536 images were used to train the fast-regional CNN detectors.

**Additional information on the procedure and setting of the detection task**

All audio files of the standardized and the experimental data sets used in this study were screened by the algorithm in the frequency range of 5 to 50 kHz, (detection parameters: analysis chunk length of 15 s, overlap of 1.5 s, for the Long detector; analysis chunk length of three seconds, overlap of 0.1 s, for the Short detector and Very short detector). The parameter analysis of chunk length specifies the length – in seconds – of the audio sections that are processed at a time (GPU-dependent), while overlap refers to the amount of overlap between audio chunks, in seconds. The values were chosen following the recommendations in Coffey et al.^25^.

**Additional information on the R-DS Filter**

The function termed “R-DS Filter” processes the output tables using the output formats, Raven (*.txt) and Excel (*.xlsx), obtained from the detection analysis on DeepSqueak. DeepSqueak allows exporting call parameter measurements and on/offset data in different formats. In contrast, the import function is more limited in the number of compatible formats from which to read data. Excel files are not supported by the import function, and Raven output can easily be manipulated and imported back again into DeepSqueak. This is the reason why this study used Excel and Raven formats to create the R-DS Filter. Both output formats slightly differ in the on- and offset values of the calls. DeepSqueak creates a measuring box around each detected call within the detection GUI. The measuring box is longer and wider than the detected call. In the Raven format, on- and offset values of the measuring boxes were stored, while in the xlsx format, the on- and offset of the calls were saved. This difference has a significant impact on the filter quality of the “R-DS Filter”, as the broader on- and offsets values within the Raven format can overlap in closely positioned syllables. As the R-DS Filter reduces duplicate syllable detections as well as merges overlapping detections into a single syllable entry, this can lead to falsely merging short calls into a bigger call when only the Raven data are used in the filtering process. In contrast, using only the xlsx data yields very narrow measuring boxes when the filtered data are uploaded again into DeepSqueak. Therefore, we recommend applying the R-DS Filter simultaneously to both formats (Raven & xlsx). Here, the xlsx data are used to filter out false positive detections, while the Raven data are used to create an output file with better measuring boxes. As .xlsx tables cannot be loaded into DeepSqueak (v 2.0), the data filtered by our R function are always saved as Raven text file (“modraven”), which can be uploaded again to DeepSqueak.

**Additional information on the procedure and setting of the classification**

The data used to train the classifier network consisted of a total of 2257 representative calls of good quality selected from the previous training data set of the detector and the good quality standardized data set. The selected vocalizations were labeled manually according to the literature^49,50^ as Long whistles (N=186), Trills (N=302), Short whistles (N=1158), Tsaks (N=541) and Zips (N=70). Afterwards, we trained the supervised classifier (setting: Frequency to pad boxed above and below each box in (kHz): 70).
